# Supplementary material for: Thiazoline-related innate fear stimuli orchestrate hypothermia and anti-hypoxia via sensory TRPA1 activation
Source: Nat Commun. 2021 Apr 6;12:2074. doi: 10.1038/s41467-021-22205-0 (PMC8024280; doi:10.1038/s41467-021-22205-0)
Supplement: Supplementary file 1 — Supplementary Information [file 41467_2021_22205_MOESM1_ESM.pdf]

Thiazoline-related innate fear stimuli orchestrate hypothermia and anti-hypoxia via sensory TRPA1 activation

Tomohiko Matsuo, Tomoko Isosaka, Yuichiro Hayashi, Lijun Tang, Akihiro Doi, Aiko Yasuda, Mikio Hayashi, Chia-Ying Lee, Liqin Cao, Natsumaro Kutsuna, Sachihiro Matsunaga, Takeshi Matsuda, Ikuko Yao, Mitsuyoshi Setou, Dai Kanagawa, Koichiro Higasa, Masahito Ikawa, Qinghua Liu, Reiko Kobayakawa, Ko Kobayakawa

Supplementary Information

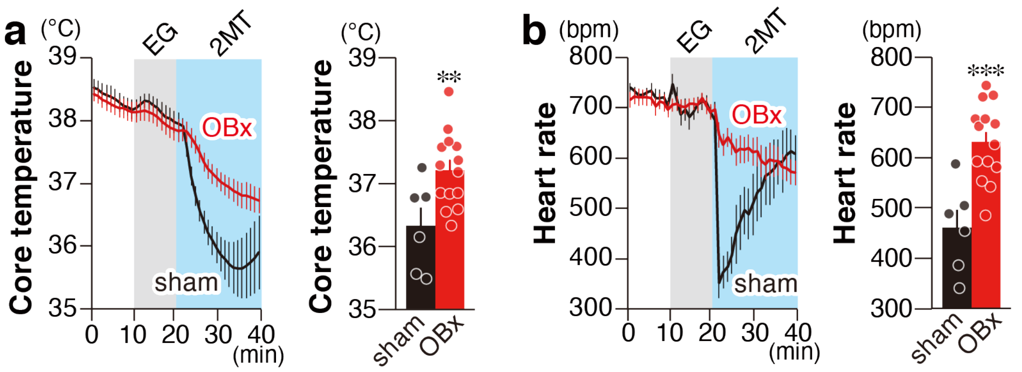

Supplementary Fig. 1 Olfactory bulb ablation suppressed 2MT-induced hypothermia and bradycardia. Core body temperature (**a**) and heart rate (**b**) in response to the consecutive presentation a filter paper containing saline (1-10 min), Eugenol (EG; 11-20 min) and 2MT (21-40 min) was analyzed for olfactory bulbectomized animals (OBx; n = 14) and sham-operated control (sham; n = 6) using implantable radio-telemetry transmitter. Mean core temperature during 2MT presentation (21-40 min) and mean heart rate during 2MT presentation (21-30 min) are shown. p = 0.0051 for core bogy temperature and p = 0.0002 for heart rate. Data are shown as mean ± SEM. Unpaired one-tailed Student's t test was used to assess significance. **$p < 0.01$, ***$p < 0.0001$

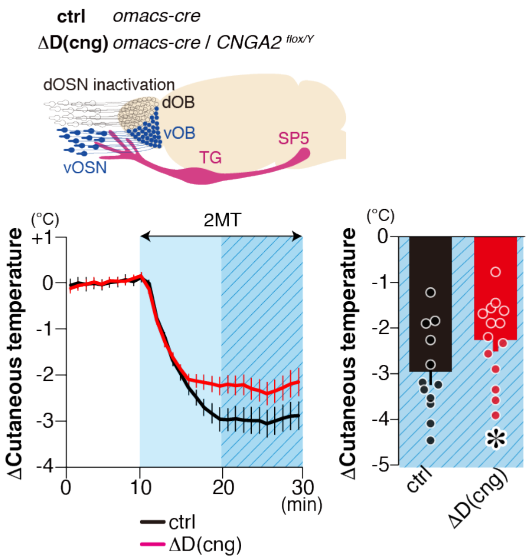

Supplementary Fig. 2 ΔD(cng) suppressed 2MT-induced hypothermia. Temporal analysis of delta cutaneous temperature of ΔD(cng) (*Omacs-Cre*; *CNGA2* $^{flox/Y}$; n = 14) and control mice (n = 12) in response to 2MT presentation are shown (left). Mean cutaneous temperature changes during 21-30 min (shaded) are also shown (right). $p = 0.0352$, unpaired one-tailed Student's t test.

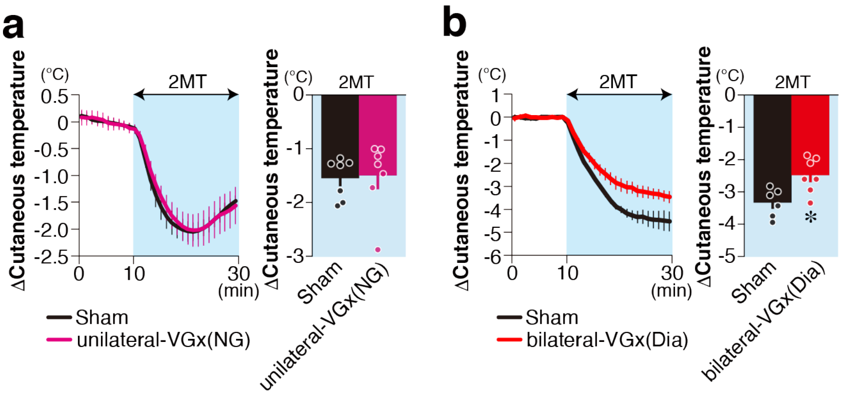

Supplementary Fig. 3 Vagus ablation suppressed 2MT-induced hypothermia. Temporal analysis of delta cutaneous temperature of sham (black; n = 7) and mice with unilateral cervical vagotomy (unilateral-VGx(NG), dark pink; n = 7) (**a**), or sham (black; n = 6) and mice with bilateral ablation of vagus nerves below the diaphragm (bilateral-VGx(Dia), red; n = 7) (**b**) in response to 2MT presentation (left). Mean cutaneous temperature changes during 20 min of 2MT presentation are also shown (right). $p = 0.2279$, one-tailed Mann-Whitney test (**a**) and $p = 0.0055$, unpaired one-tailed Student's t test (**b**). Data are shown as mean ± SEM. ** $p < 0.01$.

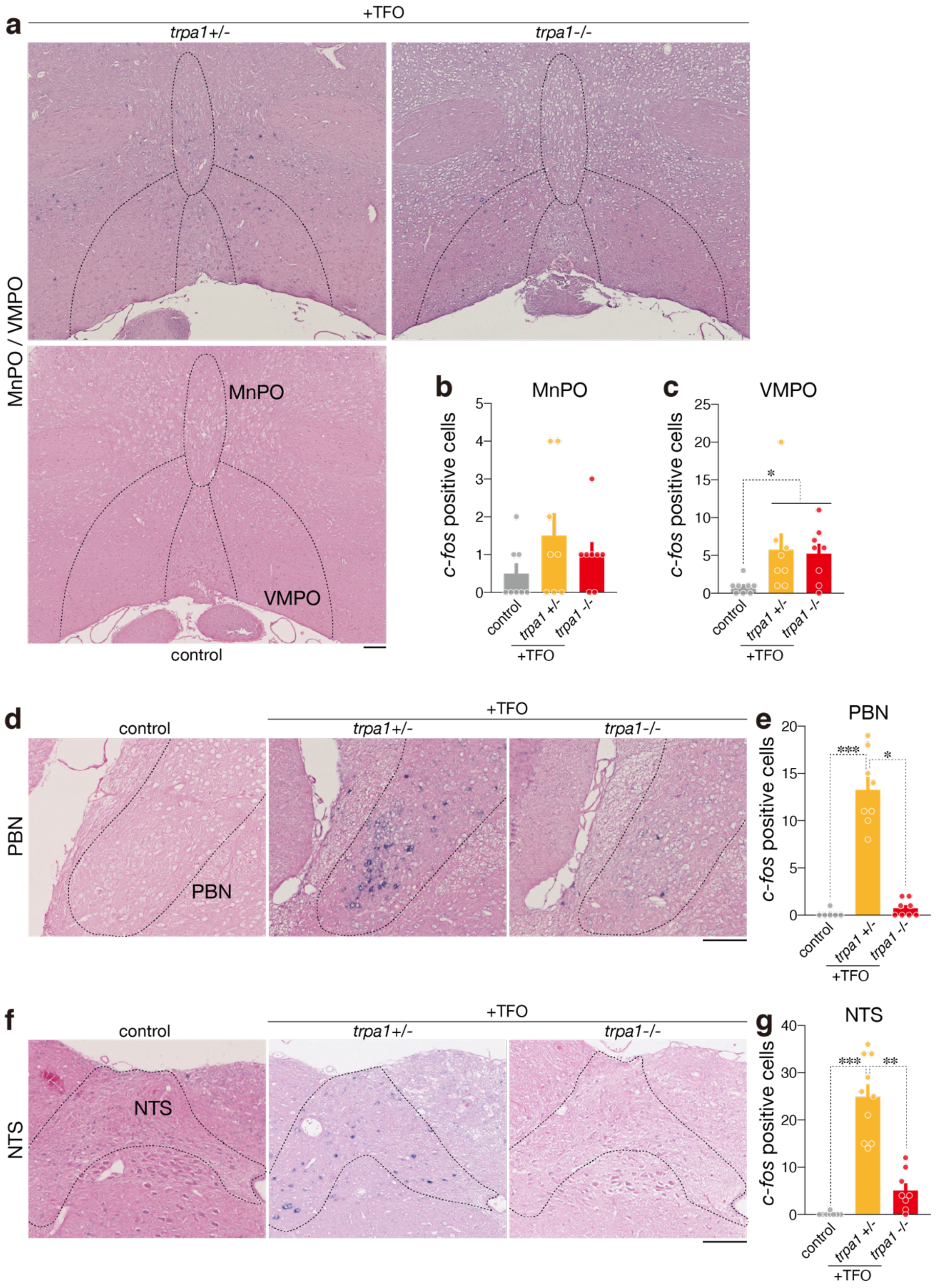

Supplementary Fig. 4 *C-fos* mRNA expression in the thermoregulatory center in the brain. *C-fos* mRNA expression in response to the presentation of filter papers containing 2MT were analyzed

for *Trpa1*$^{+/-}$ and *Trpa1*$^{-/-}$ mice by *in situ* hybridization. In the control experiment, *c-fos* mRNA expression in response to the presentation of filter papers containing saline was analyzed for wildtype animals. Representative images of the MnPO and VMPO (**a**), PBN (**d**) and NTS (**f**) are shown for each condition. Quantification of *c-fos*$^{+}$ cells are shown for MnPO (**b**; n = 8 for each, p = 0.5210 for control vs *Trpa1*$^{+/-}$, p = 0.7707 for control vs *Trpa1*$^{-/-}$ and P>0.9999 for *Trpa1*$^{+/-}$ vs *Trpa1*$^{-/-}$), VMPO (**c**; n = 8 for each, p = 0.041 for control vs *Trpa1*$^{+/-}$, p = 0.0272 for vs *Trpa1*$^{-/-}$ and $p > 0.9999$ for *Trpa1*$^{+/-}$ vs *Trpa1*$^{-/-}$), PBN (**e**; n = 8 for control and *Trpa1*$^{-/-}$ and n = 10 for *Trpa1*$^{+/-}$, $p < 0.0001$ for control vs *Trpa1*$^{+/-}$ and p = 0.2193 for control vs *Trpa1*$^{-/-}$ and p = 0.0212 for *Trpa1*$^{+/-}$ vs *Trpa1*$^{-/-}$), and NTS (**g**; n = 6 for control, and n = 8 for *Trpa1*$^{+/-}$ and *Trpa1*$^{-/-}$, p = 0.0007 for control vs *Trpa1*$^{+/-}$, $p > 0.9999$ for control vs *Trpa1*$^{-/-}$, p = 0.0048 for *Trpa1*$^{+/-}$ vs *Trpa1*$^{-/-}$).
Data are mean ± SEM. Kruskal-Wallis followed by Dunn's multiple comparison test was performed to assess significance. *$p < 0.05$; **$p < 0.01$; ***$p < 0.0001$; ns, $p > 0.05$. Scale bars, 100μm. MnPO, median preoptic nucleus; VMPO, ventromedial preoptic area; PBN, parabrachial nuclus; NTS, nucleus of the solitary tract.

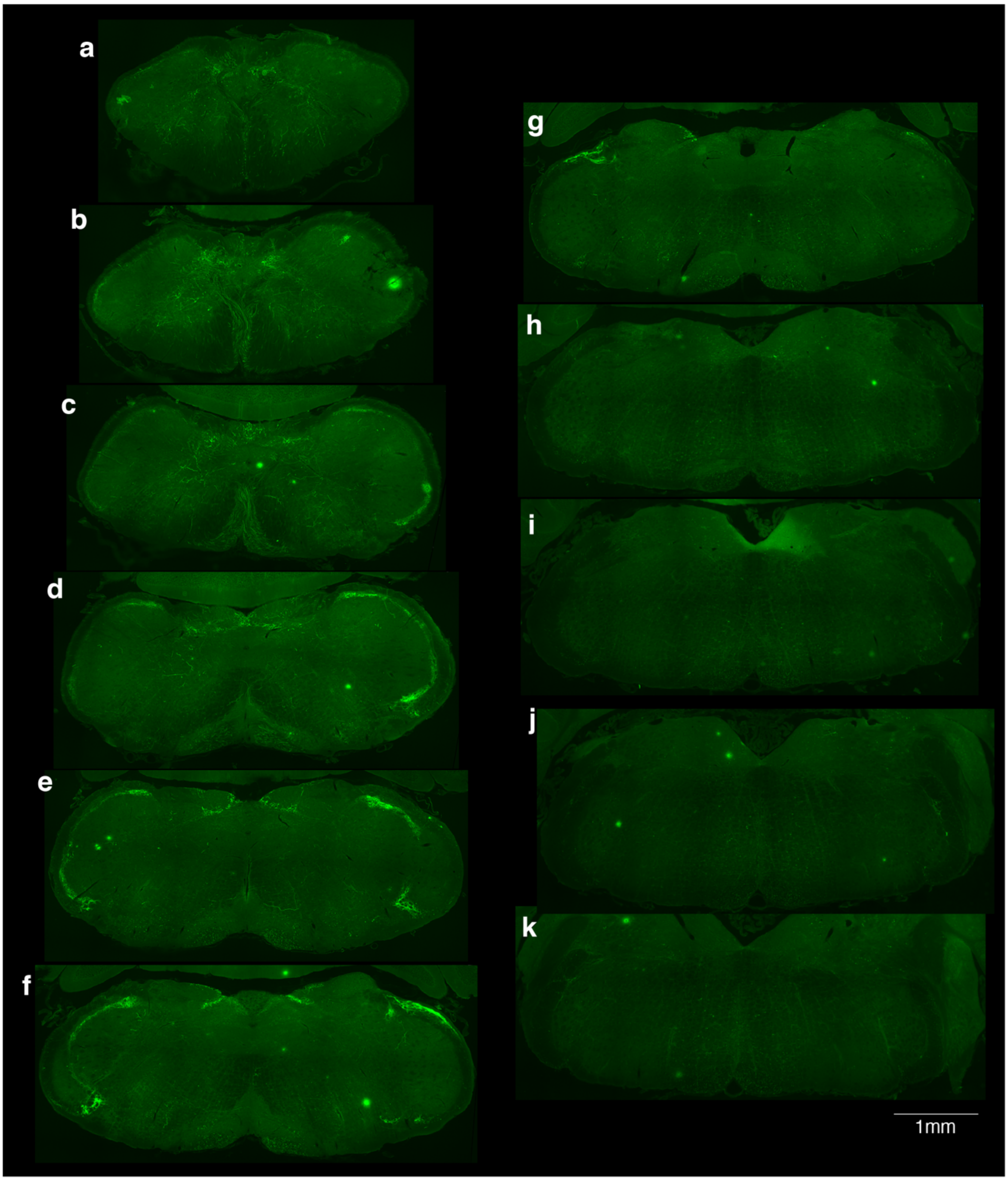

Supplementary Fig. 5 Axonal projections of *Trpa1*$^{+}$ neurons in the brainstem. Representative images of EYFP^{+} expression in serial coronal sections of the NST in *Trpa1-Cre*$^{+}$/RCL-ChR2/EYFP^{+} double transgenic mice. The sections were taken every 250 μm and arranged from caudal to rostral. EYFP^{+} fibers were observed in caudal (**a**–**f**), but not in rostral sections (**g**–**k**) of the nucleus of the solitary tract (NST).

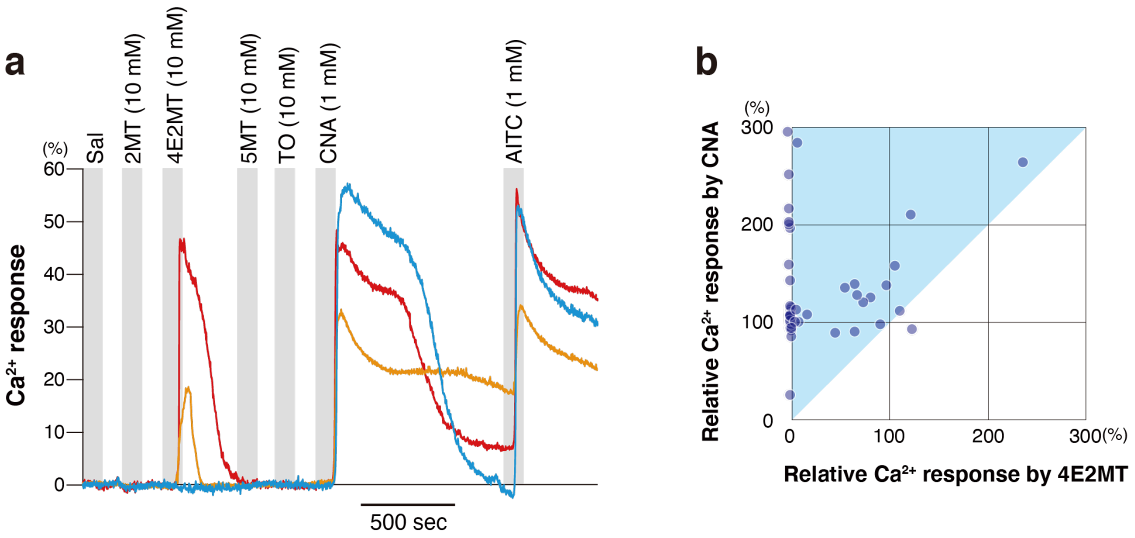

Supplementary Fig. 6 Comparison of calcium responses to CNA and 4E2MT in identical cells. **a,** Representative traces of GCaMP6f fluorescence of the VG *Trpa1*$^{+}$ neurons are shown. Examples of cell responding only to CNA (blue), that responding almost equally to CNA and 4E2MT (red), and that responding stronger to CNA than to 4E2MT (orange) are shown. **b,** For the 49 *Trpa1*$^{+}$ VG neurons that recorded calcium responses to both CNA and 4E2MT, the relative responses to CNA and 4E2MT were plotted on the XY axis. Calcium response to AITC was set to 100 % for each cell. The area shown in blue indicates cells that responded more strongly to CNA, and the area shown in white indicates cells that responded more strongly to 4E2MT.

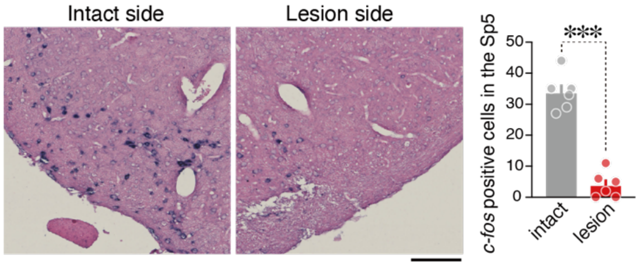

Supplementary Fig. 7 Trigeminal ganglion lesion suppressed *c-fos* expression in the ipsilateral Sp5. Representative images of Sp5 in the intact and lesion sides in the unilateral trigeminal ganglion-lesioned animal in response to 2MT presentation are shown (left). Quantification of *c-fos*$^{+}$ cells in the ventral Sp5 analyzed for intact and lesion side are also shown (right). For quantification, two sections per animal were analyzed for three independent animals (n = 6 for each side, $p < 0.0001$). Data are mean ± SEM. Unpaired one-tailed Student's *t* test. Scale bars, 100 µm.

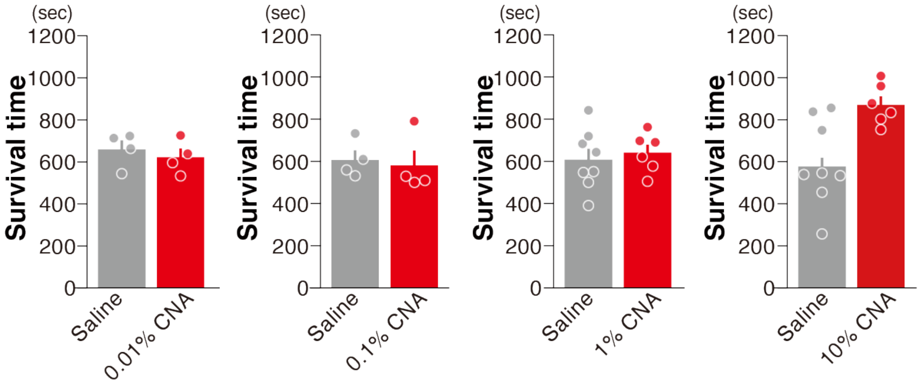

Supplementary Fig. 8 Anti-hypoxic effects of cinnamaldehyde (CNA). Mean survival time in 4% oxygen in response to the administration of 200 μl of the concentrations of CNA indicated (n = 4 for saline and 0.01% CNA, $p = 0.2699$; n = 4 for saline and 0.1% CNA, $p = 0.1714$; n = 8 for saline and n = 6 for 1% CNA, $p = 0.3169$; n = 8 for saline and n = 6 for 10% CNA, $p = 0.0001$) Only the administration of 10% CNA (approximately 800 mg/kg), higher than the LD_{50} (200 mg/kg), significantly increased the survival time in 4% oxygen. Data are shown as mean ± SEM. Unpaired one-tailed Student' *t* test was used for 0.01% CNA, 1% CNA and 10% CNA, and one-tailed Mann-Whitney U test was used for 0.1% CNA to assess significance. * $p < 0.05$; *** $p < 0.001$.

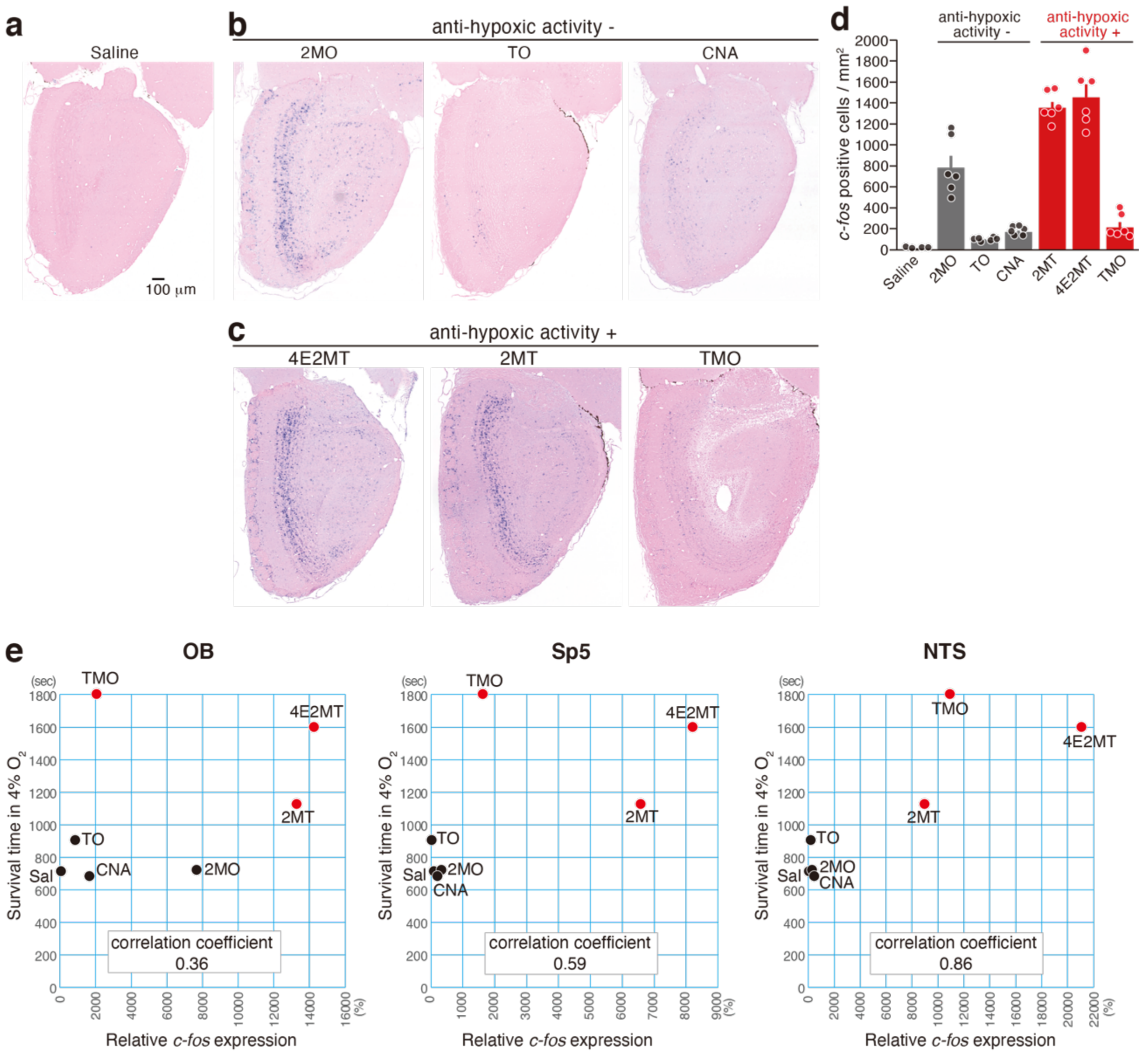

Supplementary Fig. 9 *C-fos* mRNA expression in the olfactory bulb. a Representative image of *in situ* hybridization of *c-fos* mRNA in the OB of a mouse with IP injection of Saline. **b** Representative images of *in situ* hybridization of *c-fos* mRNA in the OB of mice with IP injection of 2MO, TO, or CNA. IP injection of these compounds did not induce anti-hypoxic activity. **c** Representative images of *in situ* hybridization of *c-fos* mRNA expression in the OB of mice with IP injection of 4E2MT, 2MT or TMO. IP injection of these compounds induced anti-hypoxic activity. **d** Quantification of *c-fos* positive cells in granule cell layer in the OB (n = 4 for control, n = 6 for 2MO, TO, CNA, 2MT, 4E2MT and TMO; $p < 0.0001$ for 2MO, 2MT, and 4E2MT; $p = 0.9328$ for TO; $p = 0.4677$ for CNA; $p = 0.2624$ for TMO). Data are shown as mean ± SEM. One-way ANOVA with Dunnett’s multiple comparisons test was performed to assess significance between saline and each compound. **e** The relationship between the survival time in 4% oxygen and the number of

relative *c-fos*-expressing cells in the OB, Sp5, or NTS for each compound are shown. The vertical axis shows the survival time in 4% oxygen under IP injection of Saline (Sal), 2MO, TO, CNA, 2MT, 4E2MT or TMO. The horizontal axis shows the number of relative *c-fos*-expressing cells under IP injection of Saline (Sal), 2MO, TO, CNA, 2MT, 4E2MT or TMO. *C-fos* expression at Saline condition was set to 100%. Left OB, central Sp5, right NTS. Correlation coefficient between survival time in 4% oxygen and relative *c-fos* expression level are shown in each graph. Scale bar, 100 μm.

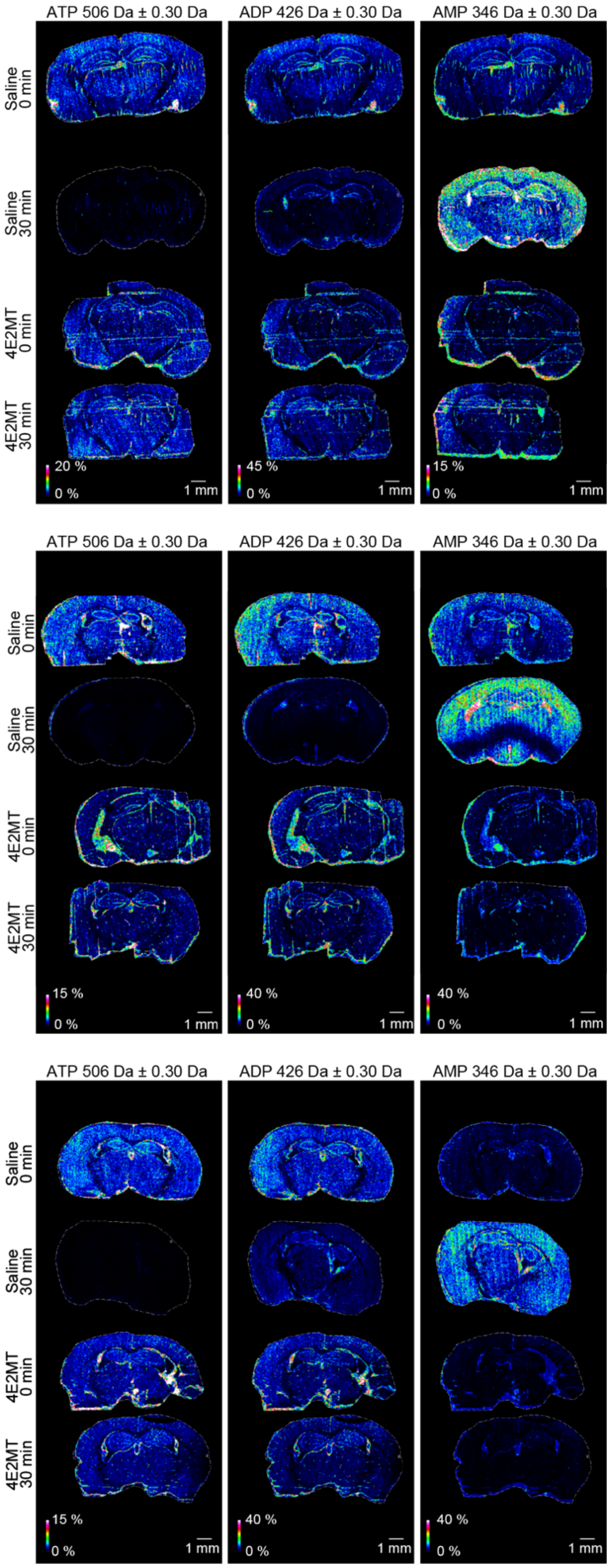

Supplementary Fig. 10 MALDI-IMS analysis of 4E2MT-treated mouse brains. Matrix-assisted laser desorption/ionization (MALDI) imaging mass spectrometry (IMS) analysis of ATP (left), ADP (middle) and AMP (right) in the coronal brain sections. Mice were IP-injected with saline or 4E2MT, and subjected to conditions with or without hypoxia (4% oxygen) for 30 min. Representative images for each treatment are shown for three individuals (top, middle and bottom columns). For control animals with saline injection, ATP and ADP were decreased, and AMP was increased after 30 min exposure to 4% oxygen. In contrast, the ATP, ADP, or AMP levels were not affected by 30 min exposure to 4% oxygen in 4E2MT-treated animals.

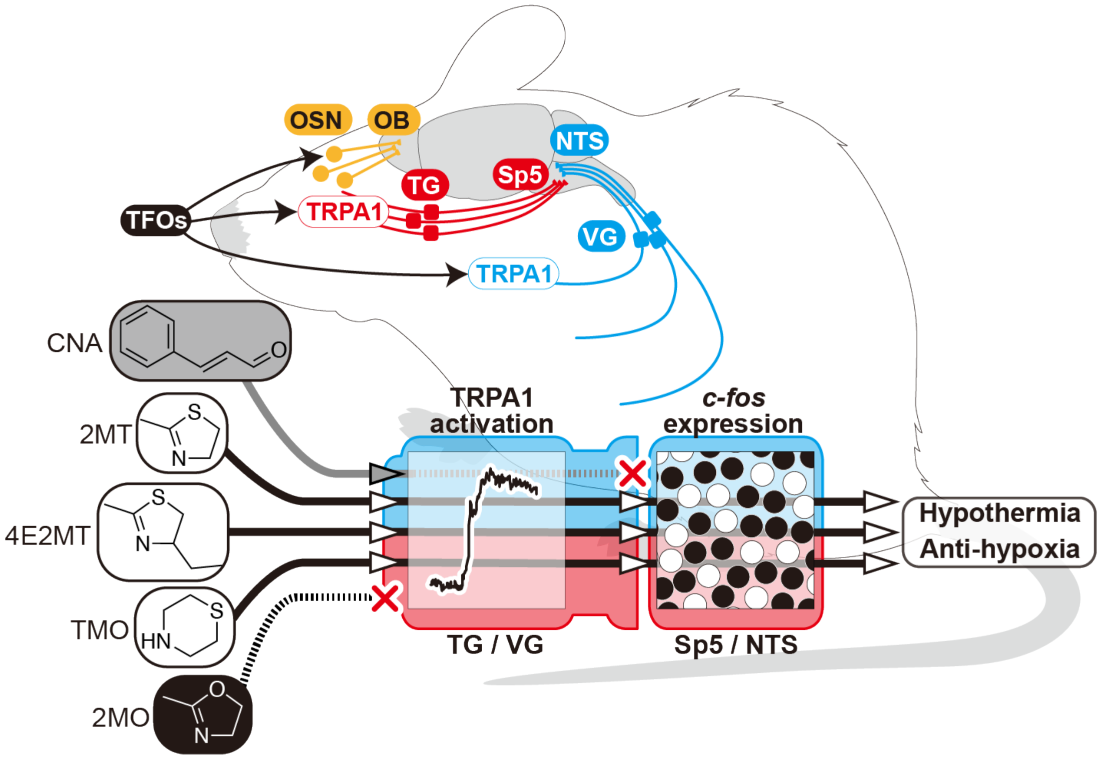

Supplementary Fig. 11 Schematic diagram of tFO–TRPA1 commanding of hypothermia and anti-hypoxia Schematic diagram of tFO–TRPA1 commanding of hypothermia and anti-hypoxia. TFOs activate at least three different sensory pathways that contribute to the regulation of innate fear induced physiological responses: olfactory sensory neuron (OSN)-olfactory bulb (OB), TG-spinal trigeminal nucleus (Sp5), and VG-nucleus of the solitary tract (NTS). Among these pathways, *Trpa1* is expressed in the TG and VG and detects tFOs. TRPA1 is activated by cinnamaldehyde (CNA), 2-methyl-2-thiazoline (2MT), 4-methyl-2-ethyl-2-thiazoline (4E2MT) and thiomorpholine (TMO), but not by 2-methyl-2-oxazoline (2MO). However, different from three other odors, CNA did not upregulate *c-fos* expression in the Sp5/NTS. To trigger hypothermia and anti-hypoxia, TRPA1 activation is not sufficient, but Sp5/NTS activation is also necessary.
