# Supplementary material for: Thiazoline-related innate fear stimuli orchestrate hypothermia and anti-hypoxia via sensory TRPA1 activation
Source: Nat Commun. 2021 Apr 6;12:2074. doi: 10.1038/s41467-021-22205-0 (PMC8024280; doi:10.1038/s41467-021-22205-0)
Supplement: Supplementary file 3 — Reporting Summary [file 41467_2021_22205_MOESM3_ESM.pdf]

nature research

Corresponding author(s): Ko Kobayakawa

Last updated by author(s): Jan 15, 2021

Reporting Summary

Nature Research wishes to improve the reproducibility of the work that we publish. This form provides structure for consistency and transparency in reporting. For further information on Nature Research policies, see our Editorial Policies and the Editorial Policy Checklist.

Statistics

For all statistical analyses, confirm that the following items are present in the figure legend, table legend, main text, or Methods section.

n/a	Confirmed	
☐	☒	The exact sample size (*n*) for each experimental group/condition, given as a discrete number and unit of measurement
☐	☒	A statement on whether measurements were taken from distinct samples or whether the same sample was measured repeatedly
☐	☒	The statistical test(s) used AND whether they are one- or two-sided<br>*Only common tests should be described solely by name; describe more complex techniques in the Methods section.*
☒	☐	A description of all covariates tested
☐	☒	A description of any assumptions or corrections, such as tests of normality and adjustment for multiple comparisons
☐	☒	A full description of the statistical parameters including central tendency (e.g. means) or other basic estimates (e.g. regression coefficient) AND variation (e.g. standard deviation) or associated estimates of uncertainty (e.g. confidence intervals)
☐	☒	For null hypothesis testing, the test statistic (e.g. *F*, *t*, *r*) with confidence intervals, effect sizes, degrees of freedom and *P* value noted<br>*Give P values as exact values whenever suitable.*
☒	☐	For Bayesian analysis, information on the choice of priors and Markov chain Monte Carlo settings
☒	☐	For hierarchical and complex designs, identification of the appropriate level for tests and full reporting of outcomes
☒	☐	Estimates of effect sizes (e.g. Cohen's *d*, Pearson's *r*), indicating how they were calculated

Our web collection on statistics for biologists contains articles on many of the points above.

Software and code

Policy information about availability of computer code

Data collection	TH92-707 data capture program 1.2 (J) (NEC Avio Infrared Technologies) for collection of cutaneous body temperature; Dataquest A.R.T. 4.30 (DSI) for collection of core body temperature and heart rate; ARCO2000 NET 3.51 (ARCO System) for collection of oxygen consumption; NDP.scan 2.5 and NDP.view2 (Hamamatsu Photonics) for imaging c-fos stained sections; LAS AF 2.7.0.9329 (Leica) and BZ-II Viewer (Keyence) for imaging fluorescent objects; Basler Pylon5 for collection of calcium imaging; FlexImaging 5.0 (Bruker Daltonics) for imaging mass spectrometry.
Data analysis	C-fos expression level was counted by single blinded investigator. Calcium imaging data was analyzed using ImageJ1.52a, and Excel 2010. RNA-seq analysis was performed using Trimmomatic v0.36, Kallisto v0.44.0, and Sleuth R package v0.30.0. Chart output of heatmap and box plots was performed using ComplexHeatmap 1.20.0 and Sleuth 0.30.0 R packages. Statistical analysis was performed with GraphPad Prism 8 and Excel for Mac (ver. 16.44).

For manuscripts utilizing custom algorithms or software that are central to the research but not yet described in published literature, software must be made available to editors and reviewers. We strongly encourage code deposition in a community repository (e.g. GitHub). See the Nature Research guidelines for submitting code & software for further information.

Data

Policy information about availability of data

All manuscripts must include a data availability statement. This statement should provide the following information, where applicable:

- Accession codes, unique identifiers, or web links for publicly available datasets
- A list of figures that have associated raw data
- A description of any restrictions on data availability

The source data underlying Figs. 1-10 and supplementary Figs. 1-8 are provided as source data files. The RNAseq data are available from https://

www.hgvd.genome.med.kyoto-u.ac.jp/repository/MGE0000001.html. The relevant data are available from the corresponding author on reasonable request.

Field-specific reporting

Please select the one below that is the best fit for your research. If you are not sure, read the appropriate sections before making your selection.

[x] Life sciences   [ ] Behavioural & social sciences   [ ] Ecological, evolutionary & environmental sciences

For a reference copy of the document with all sections, see nature.com/documents/nr-reporting-summary-flat.pdf

Life sciences study design

All studies must disclose on these points even when the disclosure is negative.

Sample size	Sample size was chosen on the basis of pilot experiments and according to previously reported publications done in the field.
Data exclusions	No data were excluded from the analysis.
Replication	Experiments were performed with sufficient animals to demonstrate statistical significance. Some experiments were replicated and similar results were obtained in Fig. 1a, b, and e; Fig. 2b; Fig. 3e; Fig. 4b-i, 5b, 6b, 10d; Supplementary Fig. 5.
Randomization	We used the mice for the experiments at random. Odor stimulation was at random in Ca2+ imaging except that CNA was applied at last because of strong induction of responses.
Blinding	Most of the data were obtained without ambiguity, automatically collected by software. The c-fos expression analysis was performed by single-blinded experimenter.

Reporting for specific materials, systems and methods

We require information from authors about some types of materials, experimental systems and methods used in many studies. Here, indicate whether each material, system or method listed is relevant to your study. If you are not sure if a list item applies to your research, read the appropriate section before selecting a response.

Materials & experimental systems

n/a	Involved in the study	
[ ]	[x]	Antibodies
[ ]	[x]	Eukaryotic cell lines
[x]	[ ]	Palaeontology and archaeology
[ ]	[x]	Animals and other organisms
[x]	[ ]	Human research participants
[x]	[ ]	Clinical data
[x]	[ ]	Dual use research of concern

Methods

n/a	Involved in the study	
[x]	[ ]	ChIP-seq
[x]	[ ]	Flow cytometry
[x]	[ ]	MRI-based neuroimaging

Antibodies

Antibodies used	Primary antibodies: rabbit anti-ERK1/2 (1:50, 16443-1-AP, Proteintech); rabbit anti-pERK(1:200, #9101, Cell Signaling Technology); rabbit anti-GFP(1:1000, ab6556, abcam); rat anti-GFP(1:1000, 04404-84, nacalai tesque). Secondary antibodies: donkey anti-rabbit conjugated with Cy3(1:800, 711-165-152, Jackson ImmunoRewearch Laboratories); goat anti-Rat IgG conjugated with Alexa Fluor 488 (1:800, 112-545-167, Jackson ImmunoResearch Laboratories); goat anti-Rabbit IgG conjugated with Alexa Fluor 488 (1:800, A11008, Invitrogen).
Validation	validation statements are on the following websites: 16443-1, https://ptglab.co.jp/products/ERK2-Antibody-16443-1-AP.htm; #9101, https://www.cellsignal.jp/products/primary-antibodies/phospho-p44-42-mapk-erk1-2-thr202-tyr204-antibody/9101; ab6556, https://www.abcam.co.jp/gfp-antibody-ab6556.html; 04404-84, https://www.nacalai.co.jp/ss/ec/EC-srchdetl.cfm?HP=1&l=EN&lc=1&syohin=0440484&syubetsu=3&catalog=&SiireC=&MakerC=&yoro=

Eukaryotic cell lines

Policy information about cell lines

Cell line source(s)	RIKEN BRC
Authentication	Authentication was performed in RIKEN by STR analysis.
Mycoplasma contamination	HEK293T cell was confirmed negative for macroplasma contamination.

Commonly misidentified lines (See ICLAC register)	No commonly misidentified cell lines were used in the study.

Animals and other organisms

Policy information about studies involving animals; ARRIVE guidelines recommended for reporting animal research

Laboratory animals	C57/BL6NCr mice; Trpa1-/- mice (JAX#006401); Trpv1-/- mice (JAX#003370); Trpa1flox mice (JAX#008649); Omp-Cre mice (JAX#006668); RCL-GCamP6f mice (JAX#028865); RCL-ChR2(H134R)/EYFP ice (JAX#024109): Trpa1-Cre mice; OMACS-Cre mice; Eno2-STO=-DTA mice; Advillin-Cre mice. All mice used were at least 9 weeks olf at the start of testing. Sex-matched control mice were used in all experiments.
Wild animals	The study did not involve wild animals.
Field-collected samples	The study did not involve samples collected from the field.
Ethics oversight	The animal experimental procedures were in accordance with the Kansai Medical University and use of laboratory animals were approved by the Animal Research Committee of Kansai Medical University.

Note that full information on the approval of the study protocol must also be provided in the manuscript.
